# Extrapolating the effect of deleterious nsSNPs in the binding adaptability of flavopiridol with CDK7 protein: a molecular dynamics approach

**DOI:** 10.1186/1479-7364-7-10

**Published:** 2013-04-05

**Authors:** C George Priya Doss, N Nagasundaram, Chiranjib Chakraborty, Luonan Chen, Hailong Zhu

**Affiliations:** 1 Medical Biotechnology Division, Centre for Nanobiotechnology, School of Biosciences and Technology, VIT University, Vellore 632014, Tamil Nadu 632014, India; 2 Department of Computer Sciences, Hong Kong Baptist University, Kowloon Tong, Hong Kong; 3 Department of Bio-informatics, School of Computer and Information Sciences, Galgotias University, Uttar Pradesh, India; 4 Key Laboratory of Systems Biology, SIBS-Novo Nordisk Translational Research Centre for PreDiabetes, Shanghai Institutes of Biological Sciences, Chinese Academy of Sciences, Shanghai, China; 5 Collaborative Research Center for Innovative Mathematical Modelling, Institute of Industrial Science, The University of Tokyo, Tokyo 153-8505, Japan

**Keywords:** nsSNPs, CDK7, Flavopiridol, Molecular dynamics, Docking

## Abstract

**Background:**

Recent reports suggest the role of nonsynonymous single nucleotide polymorphisms (nsSNPs) in cyclin-dependent kinase 7 (*CDK7*) gene associated with defect in the DNA repair mechanism that may contribute to cancer risk. Among the various inhibitors developed so far, flavopiridol proved to be a potential antitumor drug in the phase-III clinical trial for chronic lymphocytic leukemia. Here, we described a theoretical assessment for the discovery of new drugs or drug targets in CDK7 protein owing to the changes caused by deleterious nsSNPs.

**Methods:**

Three nsSNPs (I63R, H135R, and T285M) were predicted to have functional impact on protein function by SIFT, PolyPhen2, I-Mutant3, PANTHER, SNPs&GO, PhD-SNP, and screening for non-acceptable polymorphisms (SNAP). Furthermore, we analyzed the native and proposed mutant models in atomic level 10 ns simulation using the molecular dynamics (MD) approach. Finally, with the aid of Autodock 4.0 and PatchDock, we analyzed the binding efficacy of flavopiridol with CDK7 protein with respect to the deleterious mutations.

**Results:**

By comparing the results of all seven prediction tools, three nsSNPs (I63R, H135R, and T285M) were predicted to have functional impact on the protein function. The results of protein stability analysis inferred that I63R and H135R exhibited less deviation in root mean square deviation in comparison with the native and T285M protein. The flexibility of all the three mutant models of CDK7 protein is diverse in comparison with the native protein. Following to that, docking study revealed the change in the active site residues and decrease in the binding affinity of flavopiridol with mutant proteins.

**Conclusion:**

This theoretical approach is entirely based on computational methods, which has the ability to identify the disease-related SNPs in complex disorders by contrasting their costs and capabilities with those of the experimental methods. The identification of disease related SNPs by computational methods has the potential to create personalized tools for the diagnosis, prognosis, and treatment of diseases.

**Lay abstract:**

Cell cycle regulatory protein, CDK7, is linked with DNA repair mechanism which can contribute to cancer risk. The main aim of this study is to extrapolate the relationship between the nsSNPs and their effects in drug-binding capability. In this work, we propose a new methodology which (1) efficiently identified the deleterious nsSNPs that tend to have functional effect on protein function upon mutation by computational tools, (2) analyze d the native protein and proposed mutant models in atomic level using MD approach, and (3) investigated the protein-ligand interactions to analyze the binding ability by docking analysis. This theoretical approach is entirely based on computational methods, which has the ability to identify the disease-related SNPs in complex disorders by contrasting their costs and capabilities with those of the experimental methods. Overall, this approach has the potential to create personalized tools for the diagnosis, prognosis, and treatment of diseases.

## Introduction

Cyclin-dependent kinase 7 (Cdk7), a regulatory enzyme for the initiation of cell cycle progression, was initially identified from a search for cDNA encoding protein kinase(s) related to Cdk1 [1]. For activation of Cdk1, Cdk2, Cdk4, and Cdk6, the catalytic subunit of the Cdk-activating kinase requires the association of Cdk7 with a regulatory subunit, cyclin H and the phosphorylation of a conserved threonine residue at position 170 within its own T loop [2,3]. Subsequently, both CDK7 and the partner cyclin H were found to be associated with the general transcription factor TFIIH, suggesting additional roles of CDK7 in transcription. Given that CDK7 activates the main CDKs at different cell cycle transitions, it is possible to assume that the over expression of CDK7 also contributes to breast cancer cell proliferation [4]. In addition, CDK7 plays a vital role in human DNA repair mechanism (in NER pathway). Evidences support the hypothesis that mutations are early events in carcinogenesis, so the defects in DNA repair probably represent a high risk factor for many types of cancer [5,6]. Consistent with these actions, CDK7 was treated as a potent therapeutic target to inhibit the activity of cell cycle in cancerous cells. Currently, in phase-III trials for chronic lymphocytic leukemia, flavopiridol a potential antitumor drug has shown better inhibitory effect towards CDK7 [7]. It is known that flavopiridol decreases transcription by inhibiting CDK7 [8], which is responsible for the phosphorylation of the C-terminal domain of the largest subunit of RNA polymerase II, an activity essential for both transcriptional initiation and elongation [9,10]. Analyzing the human genetic variation promises to have a significant impact on the ability to understand the basis of individual variation in response to therapeutics. As we are entering the age of “personalized genomics”, it is expected that the knowledge of human genetic variations could provide a basis for understanding the differences in susceptibility to diseases and designing individualized therapeutic treatments [11,12]. It was estimated that 90% of human genetic variations were caused by single nucleotide polymorphisms (SNPs) [12]. For example, changes in amino acids of proteins, such as the nonsynonymous single nucleotide polymorphisms (nsSNPs) in the gene coding regions could account for nearly half of the known genetic variations linked to human inherited diseases [13]. The nsSNP might change the physicochemical property of a wild-type amino acid that affects the protein stability and dynamics and disrupts the interacting interface, protein-small molecule, and protein-protein interaction [14-17]. Taken together, single mutation may affect binding ability of the inhibitory molecule. Recent progress in high throughput human genome research has provided a wealth of information detailing tens of millions of human genetic variations between individuals, including SNPs [11,18]. Numerous efforts have been carried out to illustrate how nsSNPs produce deleterious effects on the stability and function of a protein [19-23]. Given the large number of SNPs, a detailed experimental study on the effect of mutation in biological function is a daunting task. An effective alternative is the use of *in silico* methods. These approaches were based on the biochemical severity of the amino acid substitution, as well as the protein sequence and/or structural information, which can provide a more feasible method for phenotype prediction.

Recently, more sophisticated *in silico* algorithms were developed to predict the impact of amino-acid substitutions on protein structure and function. Some of the variation tolerance methods follow a similar procedure, in which a missense variant is first labeled with properties, related to the damage it may cause to the protein structure or function [24]. However, in other methods, predictions are based on the difference in the free energy of unfolding (DDG) between a native-type and mutant protein. The methods that use energy functions can be subdivided into physical, statistical, and the empirical potential approaches [25]. The ultimate goal of all these approaches is to determine the deleterious nsSNPs from the neutral ones. In general, *in silico* methods can provide a feasible and the high-throughput way to determine the impact of large numbers of nsSNPs on protein function. To understand the atomistic level changes and the dynamic behavior of the molecule with respect to the potential mutations, we conducted molecular dynamics (MD) simulations analysis. MD simulations can help us understand the effects of mutation on protein structure, which allow exploring how one amino acid substitution can create a ripple effect throughout the protein structure. Offman et al. found a strong correlation between MD analysis and the experimental work on the molecular basis of the most common protein upon *N370S* mutation in causing Gaucher's disease [26,27]. Thus, we assume that MD simulation analysis might provide more reliable structural information upon CDK7 mutations.

**Figure 1:**
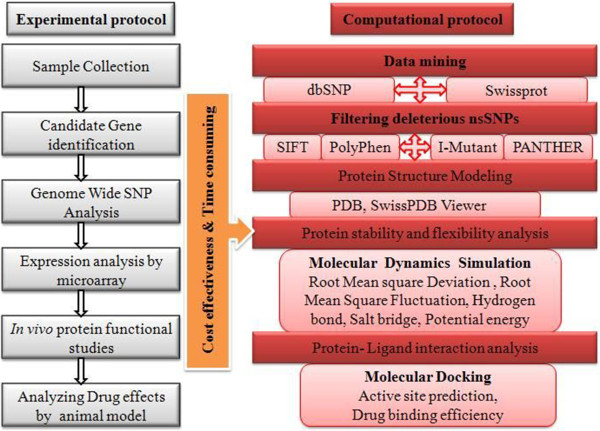
**Outline of proposed protocol for nsSNPs analysis.** This protocol explains the different steps followed in nsSNP analysis via experimental (grey color) and computational methods. Box displayed in orange color indicates the effectiveness of computational over experimental methods.

Although deleterious nsSNPs of *CDK7* gene have received considerable attention from experimental biologists, the functional consequence of most of the nsSNPs in CDK7 at the structural level is still unknown. The main goal of this *in silico* analysis is to determine the most deleterious variants in *CDK7* gene. In this context, publicly available *in silico* tools such as Sorting Intolerant From Tolerant (SIFT; J. Craig Venter Institute, Rockville, USA) [28], Polymorphism Phenotyping (PolyPhen) version 2 [29], PANTHER [30], I-Mutant3 [31], SNPs&GO (Bologna Biocomputing Group, Bologna, Spain) [32], predictor of human deleterious single nucleotide polymorphisms (PhD-SNP; Bologna Biocomputing Group) [33], and screening for non-acceptable polymorphisms (SNAP; Bologna Biocomputing Group) [34] were used to analyze the nsSNPs in *CDK7* gene. As a next step, we subjected MD simulation study in the native and mutant models of CDK7 proteins using GROMACS 4.5.3 package [35,36]. MD simulations will reveal the level of structural conformations changes with respect to the incorporation of deleterious mutations in CDK7 protein. Finally, the binding capability of CDK7 inhibitor, flavopiridol, was analyzed with respect to the structural mutations. Docking study was carried out with the help of AutoDock4 (The Scripps Research Institute, La Jolla, USA) and PatchDock [37-39]. The proposed protocol is represented schematically in Figure 1.

## Results

### Dataset

Dataset for the evaluation of potential nsSNPs in *CDK7* gene was retrieved from dbSNP [40] and SwissProt [41] database. We selected 14 nsSNPs for further consideration, and their associated biomedical informations were retrieved from OMIM (Johns Hopkins University, Baltimore, USA) [42], PubMed, and Swiss-Prot database. Related experimental data about the CDK7 protein and Protein Data Bank (PDB) structural information with PDB ID 1UA2 [43] were obtained from Swiss-Prot database and PDB, [44] respectively. The ligand molecule, flavopiridol, was obtained from Drug Bank database [45].

### Prediction of deleterious nsSNPs by *in silico* tools

Identifying the deleterious nsSNPs has become possible with the aid of improved *in silico* algorithms. Here, we analyzed 14 nsSNPs of *CDK7* gene with seven different *in silico* tools specifically SIFT, PolyPhen2, I-Mutant3, PANTHER, SNPs&GO, PhD-SNP, and SNAP to determine the protein structural and functional significance. Table 1 displays the distribution of the deleterious and neutral variations of *CDK7* gene with the corresponding amino acid substitution. SIFT makes inferences from sequence similarity using mathematical operations. SIFT constructs a multiple sequence alignment (MSA) and considers the position of the missense variants. Based on the amino acids appearing at each position in the MSA, SIFT calculates the probability and classifies a missense variant ‘tolerated’. SIFT can be applied not only to naturally occurring nsSNPs but also to identify artificial missense mutations. Among the 14 nsSNSPs analyzed by SIFT, six were identified as deleterious which obtained a SIFT score ≤0.05. PolyPhen2 utilizes a combination of sequence and structure-based attributes for the description of an amino acid substitution, and the effect of mutation is predicted by a native Bayesian classifier. The sequence-based features include position specific independent count (PSIC) scores, MSA properties, and position of mutation in relation to domain boundaries as defined by Pfam [46]. The structure-derived features are solvent accessibility, changes in solvent accessibility for buried residues, and crystallographic B-factor. By PolyPhen2, nine nsSNPs were predicted as probably and possibly damaging, having the effect on protein structure and function of CDK7 protein; the remaining five were classified as benign which obtained score less than 0.15. In order to verify the prediction accuracy of SIFT scores, we used hidden Markov model (HMM)-based evolutionary approach PANTHER to verify the effect on protein function upon a single point mutation. Out of 14 nsSNPs, 7 were designated as deleterious with a score of ≤−3. In order to improve overall prediction accuracy, we used I-Mutant3, a support vector machine-based stability prediction tool. A score less than ‘0’ means the mutation decreases the stability. The smaller the score, the more certain is the prediction. Conversely, a score more than ‘0’ means mutation increases the protein stability. Among the 14 nsSNPs of the *CDK7* gene, 13 nsSNPs showed negative DDG values, were considered to be less stable and deleterious. The remaining one nsSNP showed a positive DDG value and classified as non-deleterious. SNPs&GO is an support vector machines (SVM) classifier based on variation type and sequence environment information, sequence profiles taken from MSAs, predictions from the program PANTHER, and a function-based log-odd score describing information about protein function defined by Gene Ontology (GO) terms. SNPs&GO predicted four nsSNPs of *CDK7* gene, which are related to a disease condition. SNAP is a neural network-based method that uses *in silico* derived protein information (e.g., secondary structure, conservation, solvent accessibility, etc.) in order to make predictions regarding functionality of the mutated proteins. The network takes protein sequences and lists of mutants as input, returning a score for each substitution. These scores can then be translated into binary predictions of effect (present/absent) and reliability indices. SNAP screened four nsSNPs of *CDK7* gene as non-neutral, and it may cause phenotypic changes. PhD-SNP is a prediction method based on single sequence profile-based SVM, trained on Swiss-Prot variants. The single sequence SVM classifies the missense variant to be pathogenic or neutral, based on the nature of substitution and properties of the neighboring sequence environment. PhD-SNP classified four nsSNPs as deleterious. Comparing the results of all seven prediction tools, three nsSNPs at corresponding amino acid position I63R, H135R, and T285M with a highest SIFT tolerance index of 0.00 and PSIC score difference 1.0 were selected for structural analysis.

**Table 1:** List of nsSNPs showing deleterious/non-deleterious scores by SIFT, PolyPhen2, I-Mutant3, PANTHER SNP&GO, SNAP and PhD-SNP

**Rs IDs and variants information**	**Amino acid position**	**SIFT**	**Polyphen2**	**I-Mutant3**	**PANTHER**	**SNPs&GO**	**SNAP**	**PhD-SNP**
rs193107048	V5M	0.84	0.039	−0.7	−4.76277	Neutral	Neutral	Neutral
rs145665301	I43L	0.01	0.765	−0.58	−1.92299	Neutral	Neutral	Neutral
*rs137960738*	*I63R*	*0.00*	*1.000*	*−1.49*	*−8.80751*	*Disease*	*Non- neutral*	*Disease*
rs17849960	Q130R	0.5	0.000	0.09	−4.57361	Neutral	Neutral	Neutral
VAR_023118	G163A	0.12	0.849	−0.94	−4.76811	Neutral	Neutral	Neutral
rs201535403	N166S	0.21	0.014	−0.95	−2.03457	Neutral	Neutral	Neutral
rs180962343	V174A	0.07	0.417	−2.26	−2.27717	Neutral	Neutral	Neutral
*rs142560750*	*H135R*	*0.00*	*1.000*	*−0.02*	*−11.54699*	*Disease*	*Non-neutral*	*Disease*
rs202024894	L229W	0.00	0.98	−0.04	−4.40368	Disease	Non- neutral	Disease
rs201088666	P238L	0.00	0.997	−0.04	−2.34273	Neutral	Neutral	Neutral
rs201381439	M240I	0.12	0.11	−0.03	−1.56205	Neutral	Neutral	Neutral
*rs34584424*	*T285M*	*0.00*	*1.000*	*−0.22*	*−8.58685*	*Disease*	*Non-neutral*	*Disease*
rs200939840	R298Q	0.32	0.006	−0.63	−0.67728	Neutral	Neutral	Neutral
rs200143477	T332A	0.80	0.000	−0.23	−0.20296	Neutral	Neutral	Neutral

Highly deleterious by SIFT, PolyPhen2, I-Mutant3, PANTHER, SNPs&GO, SNAP, and PhD-SNP are italicized.

### Analysis of secondary structure and surrounding amino acid changes

**Figure 2:**
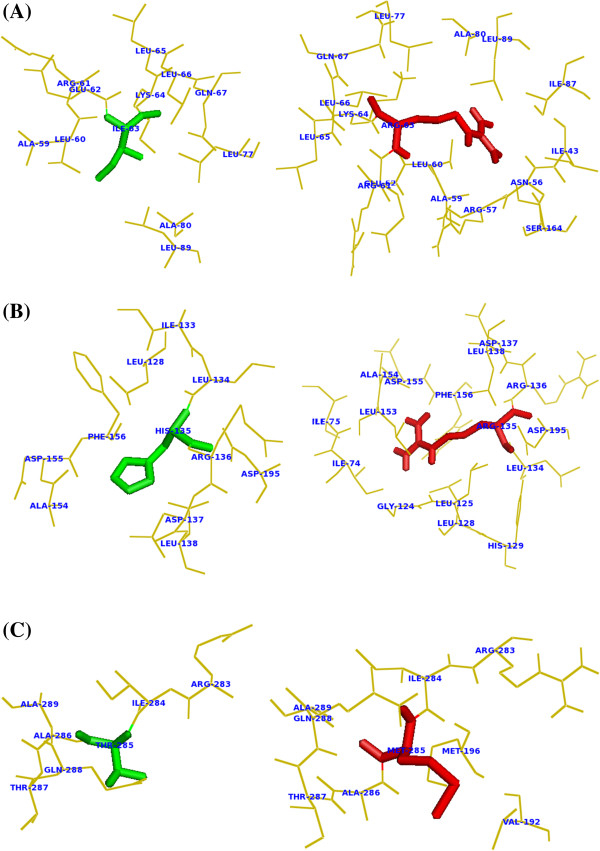
**Change in the surrounding amino acid residues in CDK7 protein by the substitution of deleterious amino acid. (A)** The native type isoleucine residue (green) at position 63 and the surrounding residues. Substitution of I63 residue with arginine (red) brings more surrounding residues in contact at position 63. **(B)** The native type histidine residue (green) at position 135 and its surrounding amino acid residues. Substitution of arginine (red) at position 135 brings more amino acids in the surrounding region. **(C)** Native type residue threonine (green) at position 285 and its surrounding amino acid residues. Substitution of methionine (red) at position 285 brings two more residues val192 and met196 within the 4 A^0^ surrounding.

Structural information could play a vital role in unveiling the molecular mechanisms leading to a disease. Based on this, we proposed modeled structures for all the three mutants (I63R, H135R, and T285M) of CDK7 protein using PyMOL (Schrödinger, Bangalore, India) [47]. Substitution of an amino acid may produce changes at the structural level. Changes in the secondary structure with respect to the substituted amino acid were analyzed in PDBsum (Cambridge, UK). Additional file 1: Figure S1 displays the secondary structural elements of the native and mutant models. The number of secondary structure elements such as beta sheets, beta hairpins, beta bulges, strands, helices, helix-helix interactions, beta turns, and gamma turns was calculated for both the native and mutant models (Table 2). It has to be noted that the observed numbers of secondary structural elements are equal in both native and mutant models except the turns. There was a slight increase in the number of beta turns in all the three mutant models. The native protein exhibited only 30, while mutants obtained 31 beta turns. Substitution of arginine in the modeled H135R protein leads to decrease in the number of gamma turns as six, whereas the native and remaining mutants I63R and T285M obtained seven gamma turns. Further, the surrounding amino acid residue changes were visualized from the point of mutational position. A residue change within 4A° surroundings was observed through PyMOL (Figure 2A–C). In addition, the number of cation-pi interacting residues for both the native and three mutants was calculated using Protein Interactions Calculator server [48]. A cation-pi interaction plays a vital role in maintaining the protein structural stability and is recognized as an important non-covalent binding interaction in structural biology [49,50]. Change in the secondary structural elements may bring about some changes in the cation-pi interacting residues in the mutant models. It has to be noted that the number of intramolecular cation-pi interactions in the native protein is seven. The substitution of deleterious amino acid increased the number of cation-pi interactions in the mutants I63R, H135R, and T285M as eight, nine, and eight, respectively (Additional file 2: Table S1). Cation-pi interacting residue distances and angles varied in mutant model showed the deleterious effects of substituted amino acid. Overall, the structural analysis results inferred that the three deleterious mutations had brought a drastic change in the CDK7 protein, and it could affect the protein function.

**Table 2:** The number of secondary structure element in the native and mutant structures of CDK7 protein

**Native and mutant models**	**Sheets**	**Beta hairpins**	**Beta bulges**	**Strands**	**Helices**	**Helix-helix interacts**	**Beta turns**	**Gamma turns**
**Native**	2	5	4	8	12	15	30	7
**I63R**	2	5	4	8	12	15	**31**	7
**H135R**	2	5	4	8	12	15	**31**	**6**
**T285M**	2	5	4	8	12	15	**31**	7

Changes in the secondary elements are italicized.

### Docking analysis

**Figure 3:**
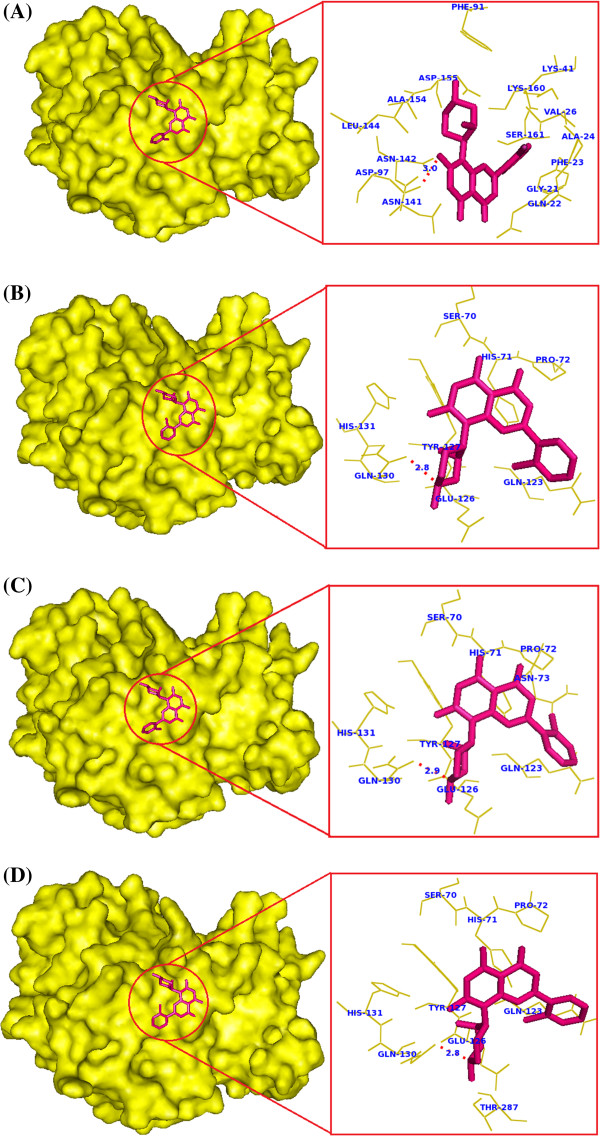
**Interaction of flavopiridol with native and mutant models of CDK7 protein. (A)** Flavopiridol binds deeply with native CDK7 protein and makes contact with 12 amino acid residues. **(B)** Substitution of I63 with arginine reduced the binding affinity of flavopiridol in mutant model I63R. **(C)** Substitution of H135 with arginine, results in weak interaction of ligand flavopiridol with H135R model. **(D)** Flavopiridol binds shallowly on the surface of mutant model T285M and the number of amino acid contact become reduced.

*In vitro* studies of flavopiridol showed inhibitory activity towards CDK7 protein and lead to programmed cell death in cancerous cells [51]. Substitution of deleterious amino acid in CDK7 protein may affect the binding ability of CDK7 with flavopiridol. This has to be analyzed to improve the potentiality of the drug to inhibit CDK7 protein. Hence, we analyzed the binding ability of flavopiridol with native and mutant models of CDK7 protein using *in silico* docking tool, Autodock4 and PatchDock [39]. Before entering into docking analysis, we evaluated the binding sites of native CDK7 protein. Flavopiridol binds at the ATP binding site of the native CDK7 protein and made contact with 12 amino acid residues. Twelve residues specifically GLY21, GLN22, PHE23, ALA24, VAL26, LYS41, PHE91, ASP97, ASN141, LEU144, ALA154, and SER161 were involved in protein-ligand interaction. This information was in concordance with the study conducted by Carlson et al. [51] and Worland et al. [52]. In their analysis, it was observed that flavopiridol directly inhibits CDK7 by competing for to the ATP binding site. In addition, these 12 residues were also involved in protein-ATP interaction observed by Lolli et al. [2] in their crystallography analysis. In the mutant models (I63R, H135R, and T285M), we observed the number of contact residues as six, seven, and six, respectively (Additional file 3: Table S2). Decrease in the number of residue contacts will definitely affect the complementarities between mutant protein and flavopiridol compound as shown in Figure 3A–D. Further, we observed the interaction of flavopiridol with native and mutant proteins by LIGPLOT (Additional file 4: Figure S2). Shape complementarity and non-covalent interactions were believed to drive protein-ligand interaction. Non-covalent bonds such as hydrogen bonds, van der Waals contacts and electrostatic forces are the dynamic forces involved in protein-ligand interactions. Calculating the interaction energies of non-covalent bonds is a key point in understanding the binding efficiency of a ligand molecule. The number of hydrogen bonds formed between protein and ligand, and van der Waals interacting energies and electrostatic interacting energies was computed using Autodock4. The binding energy and the non-covalent bond interaction energy between CDK7 protein (native and mutant) and flavopiridol molecule were calculated and shown in Table 3. In the native complex, the significant contribution of van der Waals and electrostatic energy was observed as −9.18 and −9.07 kcal/mol, respectively. On the contrary, mutant models I63R, H135R, T285M interacting with flavopiridol showed an increase in van der Waals and electrostatics energies as −5.52, −5.53, and −5.57, and −0.65, −0.6, and −0.68 kcal/mol, respectively. The total ligand receptor binding energy exhibited by the native, I63R, H135R, and T285M complexes were −8.27, −5.57, −5.53, and −5.65 kcal/mol, respectively. Lower binding energy of native complex indicates better interaction and good compatibility with the flavopiridol compound.

**Table 3:** Binding and non bonded interaction energies of native and mutant proteins of CDK7 with flavopiridol

**Proteins**	**Binding energy**	**van der Waals energy**	**Electrostatic energy**	**H-bond between protein and ligand**	**Inhibition constant**	**Inter molecular energy**	**Internal energy**	**Torsional energy**	**Ref RMS**
	**(Kcal/mol)**	**(Kcal/mol)**	**(Kcal/mol)**			**(Kcal/mol)**	**(Kcal/mol)**	**(Kcal/mol)**	
**Native**	−8.27	−9.18	−9.07	ASP97:OD1	869.56 nM	−8.86	−0.85	0.6	38.92
**I63R**	−5.57	−5.52	−0.65	GLN130:OE1	82.18 μM	−6.17	−0.7	0.6	8.55
				SER70: O					
**H135R**	−5.53	−5.53	−0.6	GLN130:OE1	87.9 μM	−6.13	−0.68	0.6	8.34
**T285M**	−5.65	−5.57	−0.68	GLN130:OE1	71.61 μM	−6.25	−0.56	0.6	8.58

In order to determine the consistency in docking analysis, in addition to Autodock4 analysis, we performed docking in PatchDock [39]. Docking was performed between the drug flavopiridol with both the native type and mutant modeled structures of CDK7 protein to find out the binding efficiency in the form of PatchDock score and atomic contact energy (ACE) values (Table 4). In this analysis, we found that native type CDK7 protein obtained high PatchDock score and ACE as 5,956 and −0.25, respectively. But, all the three mutant structures (I63R, H135R, and T285M) obtained less PatchDock scores (4,148, 4,136, and 4,978) and high ACEs (8.98, 19.92, and 21.05). Notably, high PatchDock score and less ACE value were obtained in the native complex which is considered as a good docked complex than those of other three mutant complexes. This implies the concordances of computational algorithms in docking analysis and gives a ‘theoretical quantitative’ assessment on the binding efficiency of CDK7 mutant protein with flavopiridol.

**Table 4:** PatchDock scores and ACE values of CDK7 wild type and mutant type complexes

**Native and mutant proteins**	**PatchDock score**	**Area**	**ACE Kcal/mol**
**Native**	5965	667.90	−0.25
**I63R**	4148	514.50	8.98
**H135R**	4136	594.30	19.92
**T285M**	4978	575.30	21.05

### Molecular dynamics, structural stability, and flexibility analysis

**Figure 4:**
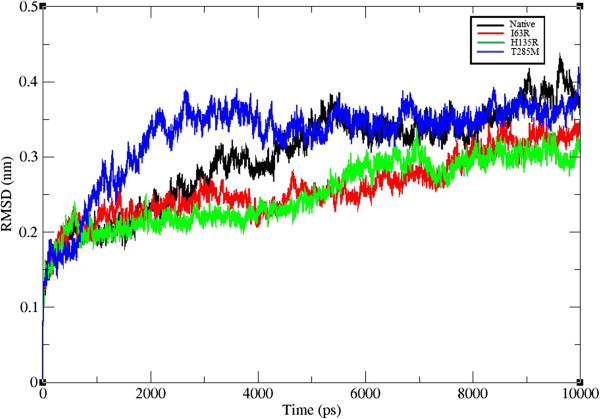
**Backbone RMSD of wild type and mutant structure of CDK7 protein.** The ordinate is RMSD (nm), and the abscissa is time (ps). Black, red, green, and blue lines indicate native, I63R, H135R, and T285M mutant structures, respectively.

**Figure 5:**
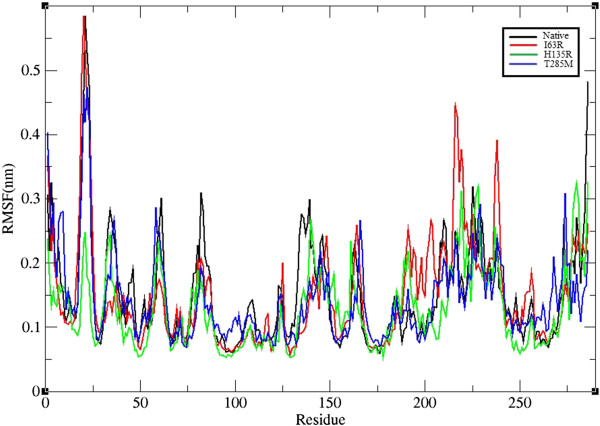
**Carbon alpha RMSF of wild type and mutant structure of CDK7 protein.** The ordinate is RMSF (nm), and the abscissa represents the residues. Black, red, green, and blue lines indicate the native, I63R, H135R, and T285M mutant structures, respectively.

Molecular dynamic simulations studies were carried out to unravel the atomic level changes in the CDK7 protein with respect to the time scale. The overall protein stability changes upon mutation were evaluated by root mean square deviation (RMSD) values. We calculated the backbone RMSD for all the atoms from the initial structure, and this is considered to be a primary criterion to measure the convergence of the protein system concerned. The backbone RMSD was calculated for both the native and mutant models from the appropriate trajectory files (Figure 4). We observed a significant structural deviation in the mutant proteins I63R, H135R, and T285M when compared to native CDK7 protein structure. All the four structures attained a significant deviation at last 5 ns. The native and T285M mutant structure obtained a mean RMSD of approximately 0.35 nm in the last 5 ns, and mutant models I63R and H135R exhibited a deviation range from about 0.25 to 0.3 nm. This difference in the deviation range in mutant model explains the stability change and reflects the impact of substituted amino acid in the protein structure. In order to determine the structural flexibility of both the native and mutant models of CDK7 protein, we calculated the root mean square fluctuation (RMSF) values from the 10 ns simulation trajectory data. The RMSF values of native and mutant models are shown in Figure 5. In the entire 10 ns simulation period, native residues from approximately 25 to 150 nm showed a high fluctuation in comparison with I63R, H135R, and T285M mutant models. In the remaining residue range from around 150 to 260 nm, the mutant model I63R exhibited high fluctuation. Overall, RMSFs of all the mutant models were significantly deviated from the native structure in the entire simulation period. A change in the RMSFs specify the mode of flexibility changes in the mutant models and reflects the impact of deleterious amino acid substitution in CDK7 protein.

### Effects of deleterious mutations in hydrogen bonding, salt bridges and electrostatic potential of CDK7 protein

**Figure 6:**
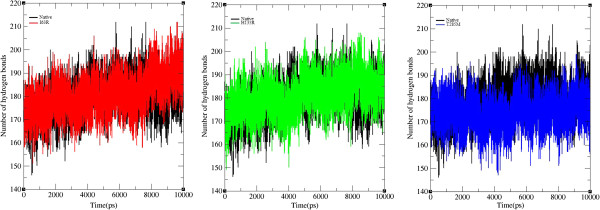
**Number of hydrogen bond formed in wild type and mutant structure of CDK7 protein.** The ordinate is the number of hydrogen bond and the abscissa is time (ps). Black, red, green, and blue lines indicate the native, I63R, H135R, and T285M mutant structures, respectively.

Hydrogen bonds and salt bridges are the key parameters in determining the stability of protein [53,54]. Non-synonymous SNPs can affect wild type protein function by affecting hydrogen bond formation [55-57]. Figure 6 depicts the number of hydrogen bonds formed in native and mutant structures of CDK7 protein. Native structure of CDK7 protein exhibits an average number of approximately 160 to 200 hydrogen bonds throughout the 10 ns simulation period. Mutant models I63R and H135R obtained more close number of hydrogen bonds, about 160 to 195 in comparison with native structure. Interestingly, T285M mutant model showed less number of hydrogen bonds around 160 to 190, when compared with the native and remaining two mutant models. Overall, it has to be noted that all the three mutant models obtained less number of hydrogen bonds in comparison with the native protein. The reduction in the number of hydrogen bonds in mutant proteins might be due to the incorporation of deleterious amino acid, and it may destroy the ability of hydrogen bond formations in CDK7 protein.

Salt bridge distances of CDK7 protein in both native and mutant proteins were calculated from the 10 ns trajectory data and shown in Additional file 5: Figure S3. In a period of about 1,000, 5,000, and 7,000 ps, native protein obtained a low salt bridge distance of approximately 0.2 nm and maintained an average range distance around 0.45 nm throughout the 10-ns simulation period. Two mutant models (I63R and H135R) maintained a similar distance like the native protein. The mutant model T285M exhibited high range of salt bridge distance in the maximum simulation period when compared to the native, I63R, and H135R structures. From this analysis, we conclude that salt bridges are more stable in I63R and H135R mutant models when compared to the mutant model T285M.

## Discussion

The central objective in molecular biology and population genetics is to identify and characterize the nsSNPs that are functionally related from those that are not. This understanding not only provides insight into cancer biology but also highlights the anticancer therapeutic targets and diagnostic markers. NsSNPs in coding region can lead to amino acid change. This can lead to alterations in protein function and account for susceptibility to disease and altered drug response. Identification of deleterious nsSNPs from tolerant nsSNPs is ideal for analyzing individual susceptibility to disease, understanding the pathogenesis of disease, identifying molecular targets for drug treatment, and conducting individualized pharmacotherapy. Several experimental studies were carried out to analyze the relationship between nsSNPs and drug response in cancer treatments. Chambers et al. [58] reported the involvement of nsSNPs in the modulation of protein structure and function. Another finding by Giovannetti et al. [59] demonstrated the role of nsSNPs in the DNA-repair protein to be the potential biomarkers of primary resistance to gemcitabine/cisplatin-based polychemotherapeutic agent in the treatment of pancreatic cancer. In another analysis, Wang and Moult [60] reported the role of nsSNPs in individuals by inducing or influencing the disease by affecting protein-protein interactions, protein expression, alternative splicing, stability, folding, and ligand binding or catalysis. These mounting studies on nsSNPs assert their role in better understanding the resultant phenotypic variations among individuals with an endeavor towards new drug design and development. The exponential increase in the number of SNPs makes the determination of biological significance of each nsSNP by wet laboratory experiments impossible. Alternatively, *in silico* programs and statistical methods may be used to predict the effects caused by mutations and elucidate the underlying biological mechanisms. However, *in silico* tools can be used to examine the potentially deleterious nsSNPs that might affect important drug targets before further investigation by wet laboratory techniques. Previously, our group also identified and analyzed the effects of deleterious nsSNPs in several proteins at structural and functional level and drug binding capability using various *in silico* tools [61-63]. In this paper, we performed a systematic *in silico* analysis to determine the potential deleterious and functional nsSNPs in CDK7 protein along with molecular dynamics and docking study. To determine the possible effects of nsSNPs in *CDK7* gene, we employed seven widely used *in silico* tools specifically SIFT, PloyPhen2, I-Mutant3, PANTHER, SNPs&GO, SNAP, and PhD-SNP. SIFT predicted 6 (42.85%) nsSNPs as deleterious, PolyPhen2 identified 9 (64.28%) substitutions which affect protein structure and function, I-Mutant3 identified 13 (92.85%) substitutions which affected the stability of protein, PANTHER predicted 7 (50%) nsSNPs to be deleterious, SNPs&GO, SNAP, and PhD-SNP identified 4 (28.57%) nsSNPs related to the disease condition. The basis for the predicting impact of nsSNPs in these seven algorithms was different, and we would expect the outcomes to occur in some ways, dissimilar. However, the positive predictions that overlap all these seven *in silico* tools would provide high reliability to behave similarly. The difference in their predictions might be due to the difference in features utilized by the methods or the training dataset. Comparing the prediction of all the seven methods, three nsSNPs (I63R, H135R, and T285M) were identified as highly deleterious and selected for further structure and functional investigations. To gain insight knowledge on the protein structure and what kind of harmful modulation these mutations give rise, the CDK7 protein was analyzed by MD approach. In the 10 ns simulation trajectory, different parameters were applied to analyze the level of structural changes. Molecular stability and flexibility changes were observed by RMSD and RMSF analyses. Stability is a fundamental property affecting the bimolecular function, activity, and regulation. Protein stability analysis results inferred that the stabilities of I63R and H135R RMSD are less deviated than those of the native and T285M protein. High or less deviation implies increase or decrease in the stability of protein. Hence, we believe that reduction in the stability of I63R and H135R models could affect the CDK7 protein structure. From the fluctuation analysis, we observed a decrease in the flexibility for all the three mutant models in first half of the residues (approximately 25 to 150) and increase in flexibility for the mutant model I63R in the rest of the residues (about 150 to 260). Increase in the flexibility could make the protein more flexible, and decrease in the flexibility makes protein more rigid. Conformational changes are required for many protein functions [64-66], but the conformational flexibility and rigidity must be well balanced [67]. The flexibility of all the three mutant models of CDK7 protein is heterogeneous in comparison with the native protein. Thus, from the RMSD and RMSF analysis, it is confirmed that substitution of amino acid adversely affected the stability and flexibility of CDK7 proteins. Beside the different electrostatic interactions, the hydrogen bonds and the salt bridges across the binding interfaces and in the protein interiors serve as main contributors in maintaining the protein structural conformation. Furthermore, incorporation of deleterious nsSNP might change the original electrostatic formations and distances that could affect the protein native structure. Consequently, CDK7 native protein obtained maximum of around 200 hydrogen bonds in the 10 ns simulation period. The mutant models I63R, H135R, and T285M obtained less hydrogen bonds approximately 195 and 190, respectively. The decrease in the number of hydrogen bonds may affect the protein structure. In salt bridge analysis, both the native and mutant models of CDK7 protein maintained the different patterns of salt bridge distances. Changes in the salt bridge distances reflect the displacement of cationic or anionic side chain residues in mutant models. In conclusion, we observed change in bonding distance by hydrogen bonding and salt bridge analysis. Change in residue distance might lead to the loss of thermodynamic stability. The main aim of this study is to extrapolate the relationship between the nsSNPs and their effects in drug-binding capability. In docking analysis, several factors involved between protein-ligand interactions were analyzed, and the analysis revealed the less binding ability of mutant models. In particular, electrostatic potential showed substantial agreement with MD analysis. In conclusion, the given *in silico* tools can indicate possible deleterious nsSNPs in CDK7 protein. Then, MD studies support the structural and conformational changes for the CDK7 deleterious nsSNP incorporated model. Finally, the binding ability of mutant model with the drug was validated to facilitate the study of new drug-targets and discovery of new drugs for CDK7 protein. *In silico* approaches reviewed here generated not only a considerable amount of valuable data but also the need for further validation by experimental methods such as *in vitro* binding/activity assays.

## Materials and methods

### Computational methods for finding deleterious variants

The ability to distinguish pathogenic and benign variants from a pool of data is a daunting task. Recently, many computational algorithms have been developed for the feasible prediction of disease-associated variants. Some of the methods classify deleterious variants according to the predicted pathogenicity, and other methods predict the deleterious variants based on protein-stability changes upon mutation. We used both these approaches to identify deleterious variants in the *CDK7* gene. Sequence evolutionary information-based methods (SIFT, PANTHER, and PhD-SNP) and the combination of protein structural and functional parameter-based methods (PolyPhen2, I-Mutant3, SNAP, and SNPs&GO) are some of the most reliable tools used for deleterious nsSNP prediction. SIFT, PANTHER, PhD-SNP, SNAP, SNPs&GO, and I-Mutant3 give results in two prediction categories, either tolerated or deleterious, while PolyPhen2 gives results in three categories: benign (probably lacking any phenotypic effect), possibly damaging, and probably damaging (should affect protein function). Sequence-based prediction includes all types of effect at the protein sequence level and can be applied to any human protein with known relatives. Structure-based approach is feasible to implement for proteins with 3D structures. Analyzing deleterious nsSNPs by both sequence and structure level has the added advantage of being able to assess the reliability of the generated prediction results by cross-referencing the results from both approaches. SIFT predicts whether an amino acid substitution affects protein function based on sequence homology and the physical properties of amino acids. A SIFT score ≤0.05 indicates that the amino acid substitution is intolerant or deleterious, whereas a score ≥0.05 is predicted as tolerant [68,69]. PANTHER estimates the likelihood of a particular nsSNP causing a functional impact on the protein. PANTHER uses HMM-based statistical modeling methods and multiple sequence alignments to perform evolutionary analysis of coding nsSNPs. PANTHER subPSEC scores vary from 0 (neutral) to about −10 (more likely to be deleterious). Protein sequences having subPSEC values ≤−3 is said to be deleterious. PolyPhen2.0 uses sequence, phylogenetic, and structural information in characterizing the deleterious substitution. A mutation is classified as ‘probably damaging’ if the probabilistic score is above 0.85 to 1; mutation is classified as ‘possibly damaging’ if the probabilistic score is above 0.15 to 0.84; the remaining mutations are classified as benign. I-Mutant3 is an SVM-based method for the automatic prediction of protein stability changes upon a single point mutation. The output file shows the predicted free energy change (DDG) which is calculated from the unfolding Gibbs free energy change of the mutated protein minus the unfolding free energy value of the native protein (Kcal/mol). DDG >0 means that the mutated protein has high stability and vice verse. PhD-SNP is a single sequence SVM method (SVM sequence) that discriminates disease-related mutations based on the local sequence environment of the mutation at hand and a sequence-profile-based SVM. The tool aims to predict whether an nsSNP causing a single point protein mutation would be a neutral polymorphism or one that is deleterious. SNPs&GO is a method based on SVMs, which predicts disease-associated mutations from protein sequence, evolutionary information, and functions as encoded in the gene ontology terms. The use of functional GO terms is the main aspect of novelty of this tool over other existing bioinformatics tools. From the output of the programs, we only took the binary prediction (pathogenic/neutral) into consideration without taking into account any confidence values provided by some of the programs. SNAP is based on neural network and advanced machine-learning approach to predict the functional effects of nsSNPs in proteins. It uses sequence, functional and structural (secondary structure, solvent accessibility) annotations, and biophysical and evolutionary (residue conservation within sequence families) characteristics to predict a gain or loss in protein function. SNAP predicts whether the mutation is neutral or non-neutral with expected accuracy.

### Protein-ligand docking analysis

Protein-ligand interaction study was performed between the native and mutant models of CDK7 protein with the inhibiting compound, flavopiridol. In order to carry out the docking analysis, we used the AutoDock4 suite as a molecular-docking tool. AutoDock4 is a suite of programs making it possible to predict how ligands bind to large macromolecules. In this docking simulation, we used semi-flexible docking protocols. Throughout the docking simulation, the target protein is kept rigid. The ligand being docked is usually flexible and, therefore, explores an arbitrary number of torsional degrees of freedom in addition to the six spatial degrees of freedom spanned by the translational and rotational parameters. AutoDock4 provides different optimization algorithms to search the space of possible protein-ligand combinations, such as simulated annealing, genetic algorithm (GA), and hybrid evolutionary algorithms EA termed Lamarckian GA (LGA) combining the GA with a local search strategy [70]. The Lamarckian Genetic Algorithm (LGA) was chosen to search for the best conformers. The best docking solution (minimum docked free energy) is reported by AutoDock for each GA run. The total number of clusters and the rank of each docking mode (cluster rank) are also reported in the cluster analysis performed by AutoDock. Docking modes were selected on the basis of two criteria: extent of ligands associations with the key residues of the receptor and the thermodynamic stability of the docked complex so obtained. The lowest energy docking mode that would conform to the above said two parameters was selected from over 10 GA runs and hence 10 total docking mode times. The grid boxes were centered on the root of macromolecule with spacing of 0.375 Å. The estimated binding free energies were calculated using the following equation: *E*_binding_ = *E*_intermolecular_ + *E*_internal_ + *E*_torsional_ − *E*_unbound_. The unbound structure of ligand is the same as the bound state (crystal structure), so the *E*_internal_ is equal to *E*_unbound_, and they do not contribute to the total energy. On the other hand, the *E*_torsional_ is calculated based only on the number of torsional bond in ligand, so this term remains the same in each complex. It is clear that there are significant differences between nine charge methods in the estimated binding free energies, so the difference should come from the *E*_intermolecular_, including energies of dispersion/repulsion, hydrogen bonding (hbond), desolvation potential, and electrostatic interactions. The energy functions used in docking simulations attempt to account for the intermolecular energies between the protein and the ligand, as well as the intramolecular energies arising from the ligand conformation itself. AutoDock4 uses a grid-based approach to approximate the energy calculations used by the energy function. During the evaluation of a candidate conformation, the grids were used as lookup tables which store the values used in the calculation, thus making the overall docking simulation exceptionally fast. The Graphical User Interface program ‘Auto Dock Tools’ was used to prepare, run, and analyze the docking simulations. Kollman united atom charges, solvation parameters, and polar hydrogens were added into the receptor PDB information for the preparation of protein in docking simulation. Gasteiger charges were added in the ligand PDB file.

In addition to the Autodock4 study, we used PatchDock for docking native and mutant CDK7 proteins with the drug flavopiridol. PatchDock performs docking based on molecular shape representation and surface patch matching plus filtering and scoring. PatchDock is more reliable because of its fast transformational search, which is driven by local feature matching rather than brute force searching for the six-dimensional transformation space. It further speeds up the computational processing time by utilizing advanced data structures and spatial pattern detection techniques, such as geometric hashing and pose clustering. Protein and the ligand molecule were given as input in performing the docking experiments with default root-mean-square deviation (RMSD) value (4.00 Å). It generated several complex structures based on docking scores. The complex structure file, with the best docking score was selected for further analysis. The geometry of both wild type and mutant type CDK7 structures were optimized through Steepest Descent method with 1000 steps each of GROMACS 4.5.3 package. Each minimization was carried out with GROMOS-96 [71] 43a1 force field.

### Molecular dynamics simulation protocol

Molecular dynamics simulations for the native and mutant models were done with MD simulation package GROMACS 4.5.3 that adopts GROMOS96 43a1 force field parameter for energy minimizations. Energy minimized structures of the native CDK7 and three mutant models were used as a starting point for MD simulations. All the proteins were solvated in a cubic box with wall extending at least 0.9 nm from all atoms and filled with SPC [72] water molecules. A periodic boundary condition was applied that the number of particles, pressure and the temperature were kept constant in the system. In order to obtain electrically neutralized system, we utilized GENION procedure from the GROMACS package to replace random water molecule with Na^+^ or Cl^-^ ions. The temperature was kept constant by using a Berendsen algorithm [73] with a coupling time of 0.2. The minimized system was equilibrated for 10,000ps each at 300 K by position restrained molecular dynamics simulation in order to soak the water molecules into the macromolecules. The equilibrated systems were then subjected to molecular dynamics simulations for 10 ns each at 300 K. In all simulations, the temperature was kept constant at 300 K. The particle mesh Ewald method [74] was used to treat long-range Coulombic interactions and the simulations performed using the SANDER module [75]. The SHAKE algorithm was used to constrain bond lengths involving hydrogen's permitting a time step of 2fs. The coordinates were saved at regular time intervals of 1ps. The van der Waals force was maintained at 1.4 nm, and Coulomb interactions were truncated at 0.9 nm.

### Analysis of molecular dynamics trajectories

Structural properties of the native and mutant models of CDK7 protein were calculated from the trajectory files with the built-in functions of GROMACS 4.5.3 The trajectory files were analyzed through the use of g_rmsd and g_rmsf GROMACS utilities in order to obtain the RMSD and RMSF values. The number of distinct hydrogen bonds formed in the protein during the simulation was calculated using g_hbond utility. The number of hydrogen bond was determined on the basis of donor-acceptor distance less than 3.9 nm and of donor-hydrogen-acceptor angle larger than 90° [76]. Salt bridge formed in CDK7 protein was analyzed using g_salt GROMACS. If the distance is ≤4.0 nm, the pair is counted as a salt bridge [77]. In order to generate the three-dimensional backbone RMSD, RMSF of carbon alpha-carbon, hydrogen bond and salt bridge analysis, and motion projection of the protein in phase space of the system were plotted for all four simulations using Graphing, Advanced Computation and Exploration program.

## Abbreviations

ACE: Atomic contact energy; CDK7: Cyclin-dependent kinase 7; SNPs: Single Nucleotide Polymorphisms; nsSNPs: Nonsynonymous Single Nucleotide Polymorphisms; GRACE: Graphing Advanced Computation and Exploration; MSA: Multiple Sequence Alignment; RMSD: Root mean square deviation; RMSF: Root mean square fluctuation.

## Competing interests

The authors declare that they have no competing interests.

## Authors’ contributions

NN, CGPD, CC, HZ, and LC were involved in the design of the study, drafting the manuscript, and the acquisition, analysis, and interpretation of the data. HZ, CC, and LC supervised the entire study. CGPD, CC, and HZ were involved in the final drafting of the manuscript. All authors read and approved the final manuscript.

## Supplementary Material

Additional file 1: Figure S1 Secondary structural elements changes in mutant models of CDK7 protein. **(A)** Secondary structural elements of native CDK7 protein. **(B)** Secondary structural element changes in the mutant model I63R due to the substituted of arginine. **(C)** Substitution of histidine at position 135 showing the secondary structural changes in H135R mutant model. **(D)** Substitution of methionine at position 285 showing the secondary structural changes in T285M mutant model. All the substituted amino acids are indicated by green boxes. Click here for file

Additional file 2: Table S1 Involvement of cation-Pi interaction in wild type and mutant structure of CDK7 protein. Click here for file

Additional file 3: Table S2 Flavopiridol interacting residues with CDK7 wild type and mutant type structures. Click here for file

Additional file 4: Figure S2 LIGPLOT analysis of CDK7-flavopiridol complex in both native and mutant states. (**A**) Native complex showing high number of residues interacting with ligand. **(B)** LIGPLOT showing interaction between mutant model I64R and flavopiridol. (**C**) LIGPLOT showing interaction between mutant model H135R and flavopiridol. **(D)** LIGPLOT showing interaction between mutant model T285M and flavopiridol. Click here for file

Additional file 5: Figure S3 Salt bridge forming distances of wild type and mutant structures of CDK7 protein. The ordinate is distance (nm) and the abscissa is time (ps). Black, red, green, and blue lines indicate native, I63R, H135R, and T285M structures, respectively. Click here for file
