# Supplementary figures and images for: Extrapolating the effect of deleterious nsSNPs in the binding adaptability of flavopiridol with CDK7 protein: a molecular dynamics approach
Source: Hum Genomics. 2013 Apr 5;7(1):10. doi: 10.1186/1479-7364-7-10 (PMC3726351; doi:10.1186/1479-7364-7-10)

**Figure S1**

**
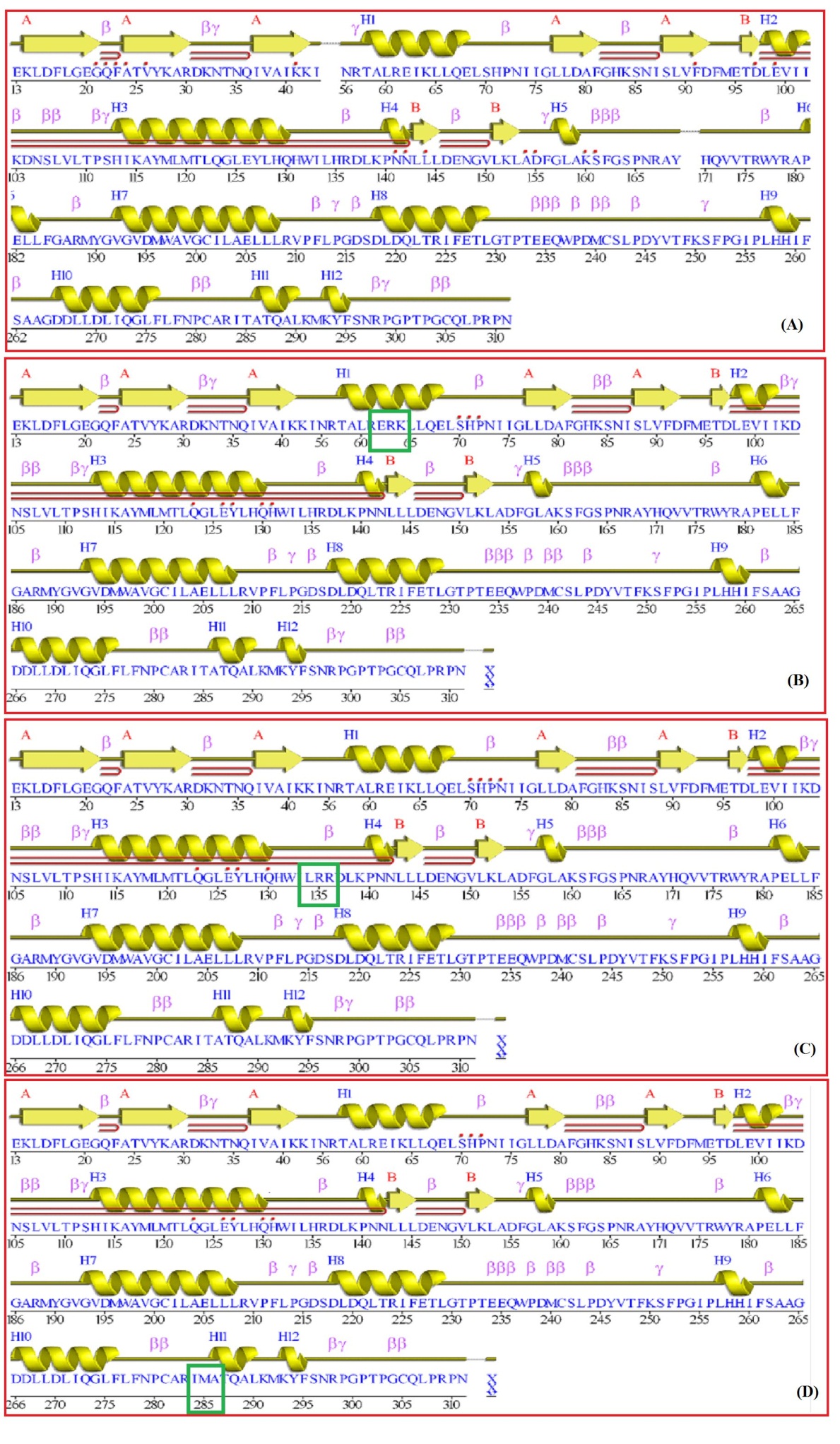
**

Supplement: Additional file 1: Figure S1 — Secondary structural elements changes in mutant models of CDK7 protein. (A) Secondary structural elements of native CDK7 protein. (B) Secondary structural element changes in the mutant model I63R due to the substituted of arginine. (C) Substitution of histidine at position 135 showing the secondary structural changes in H135R mutant model. (D) Substitution of methionine at position 285 showing the secondary structural changes in T285M mutant model. All the substituted amino acids are indicated by green boxes. [file 1479-7364-7-10-S1.doc]

**Figure S2**


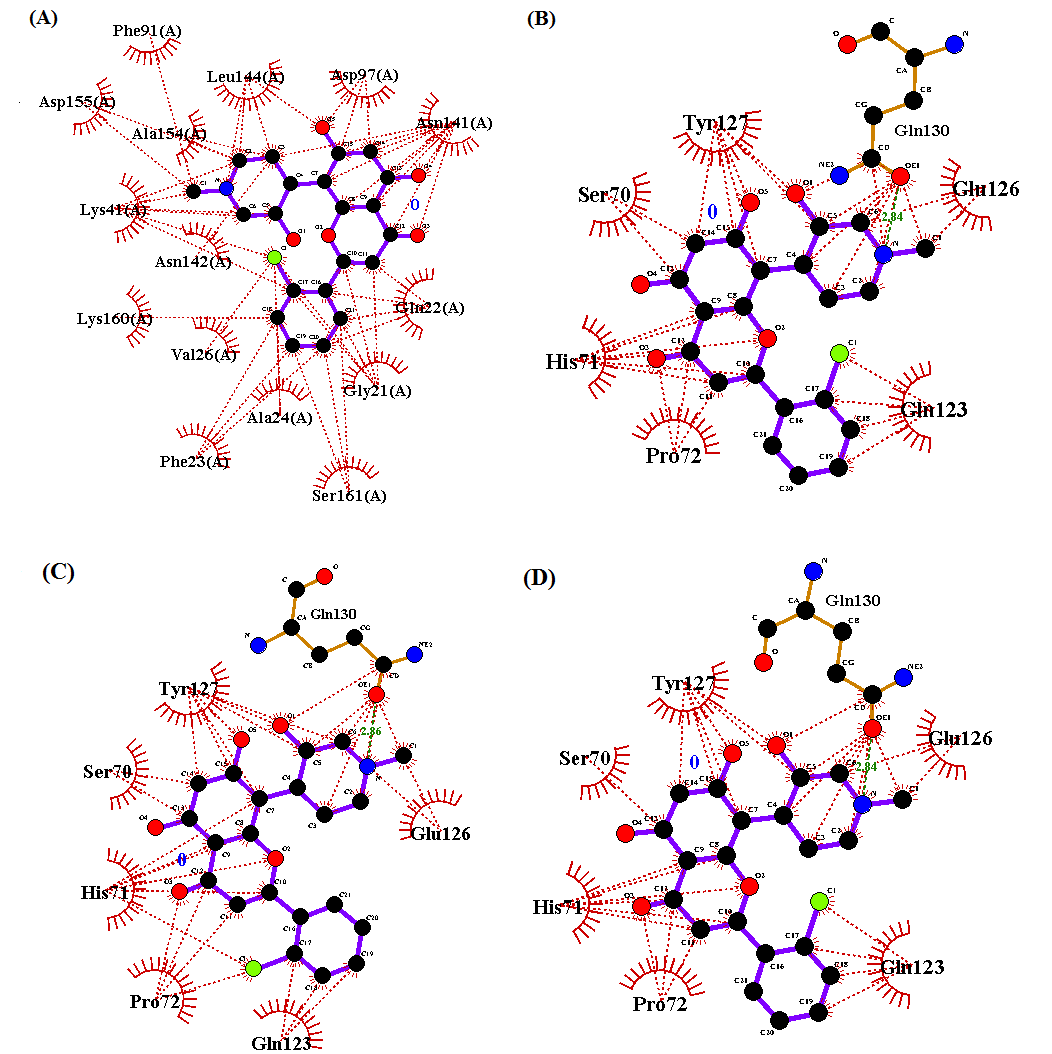

Supplement: Additional file 4: Figure S2 — LIGPLOT analysis of CDK7-flavopiridol complex in both native and mutant states. (A) Native complex showing high number of residues interacting with ligand. (B) LIGPLOT showing interaction between mutant model I64R and flavopiridol. (C) LIGPLOT showing interaction between mutant model H135R and flavopiridol. (D) LIGPLOT showing interaction between mutant model T285M and flavopiridol. [file 1479-7364-7-10-S4.doc]

**Figure S3**


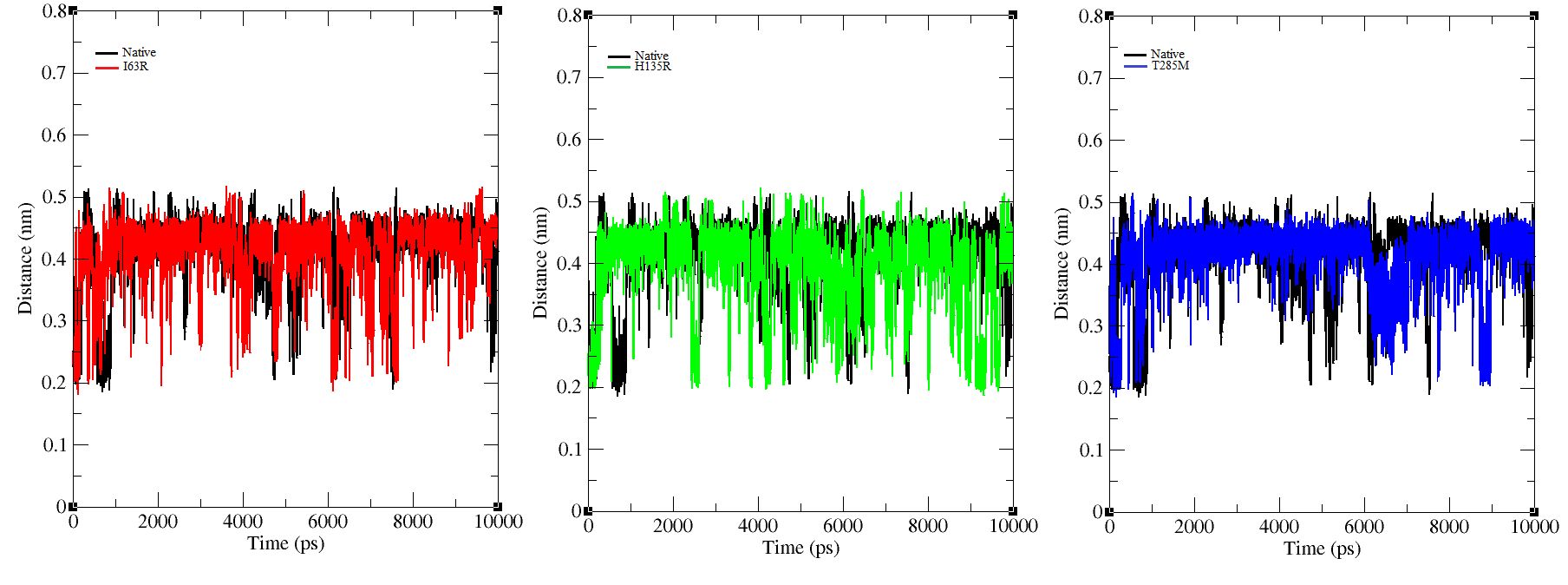

Supplement: Additional file 5: Figure S3 — Salt bridge forming distances of wild type and mutant structures of CDK7 protein. The ordinate is distance (nm) and the abscissa is time (ps). Black, red, green, and blue lines indicate native, I63R, H135R, and T285M structures, respectively. [file 1479-7364-7-10-S5.doc]
