# Supplementary material for: Extrapolating the effect of deleterious nsSNPs in the binding adaptability of flavopiridol with CDK7 protein: a molecular dynamics approach
Source: Hum Genomics. 2013 Apr 5;7(1):10. doi: 10.1186/1479-7364-7-10 (PMC3726351; doi:10.1186/1479-7364-7-10)
Supplement: Additional file 2: Table S1 — Involvement of cation-Pi interaction in wild type and mutant structure of CDK7 protein. [file 1479-7364-7-10-S2.doc]

**Supplementary Table S1.** Involvement of Cation-Pi interaction in native and mutant model of CDK7 protein.

| **CDK7 native and mutant model** | **Aromatic Residue**  **position** | **Aromatic**  **Residue**  **Name** | **Cation Residue**  **Position** | **Cation Residue** | **Distance** | **Angle** |
| --- | --- | --- | --- | --- | --- | --- |
| **Native** | 23 | PHE | 167 | ARG | 5.59 | 62.71 |
|  | 27 | TYR | 14 | LYS | 4.31 | 155.27 |
|  | 91 | PHE | 41 | LYS | 5.17 | 136.29 |
|  | 93 | PHE | 28 | LYS | 5.00 | 145.44 |
|  | 190 | TYR | 136 | ARG | 4.59 | 129.68 |
|  | 197 | TRP | 283 | ARG | 4.96 | 111.68 |
|  | 294 | TYR | 115 | LYS | 3.77 | 28.45 |
| **I63R** | 23 | PHE | 167 | ARG | 5.33 | 59.53 |
|  | 27 | TYR | 14 | LYS | 4.10 | 158.36 |
|  | 81 | PHE | 32 | LYS | 5.66 | 143.87 |
|  | 91 | PHE | 41 | LYS | 5.27 | 145.05 |
|  | 93 | PHE | 28 | LYS | 4.91 | 154.30 |
|  | 190 | TYR | 136 | ARG | 4.47 | 131.11 |
|  | 197 | TYR | 283 | ARG | 4.79 | 114.35 |
|  | 294 | TYR | 115 | LYS | 3.54 | 29.79 |
| **H135R** | 23 | PHE | 167 | ARG | 5.27 | 56.23 |
|  | 27 | TYR | 14 | LYS | 4.07 | 157.81 |
|  | 81 | PHE | 32 | LYS | 5.63 | 144.68 |
|  | 91 | PHE | 41 | LYS | 5.21 | 146.79 |
|  | 93 | PHE | 28 | LYS | 5.00 | 158.62 |
|  | 156 | PHE | 135 | ARG | 5.87 | 93.64 |
|  | 190 | TYR | 136 | ARG | 4.49 | 131.10 |
|  | 197 | TRP | 283 | ARG | 4.79 | 115.12 |
|  | 294 | TYR | 115 | LYS | 3.54 | 31.06 |
| **T285M** | 23 | PHE | 167 | ARG | 5.30 | 58.06 |
|  | 27 | TYR | 14 | ARG | 4.10 | 158.39 |
|  | 81 | PHE | 32 | LYS | 5.67 | 144.57 |
|  | 91 | PHE | 41 | LYS | 5.20 | 144.87 |
|  | 93 | PHE | 28 | LYS | 5.03 | 156.61 |
|  | 190 | TYR | 136 | ARG | 4.48 | 130.41 |
|  | 197 | TYR | 283 | ARG | 4.77 | 115.09 |
|  | 294 | TYR | 115 | LYS | 3.57 | 30.95 |
|  |  |  |  |  |  |  |
