# Supplementary material for: Extrapolating the effect of deleterious nsSNPs in the binding adaptability of flavopiridol with CDK7 protein: a molecular dynamics approach
Source: Hum Genomics. 2013 Apr 5;7(1):10. doi: 10.1186/1479-7364-7-10 (PMC3726351; doi:10.1186/1479-7364-7-10)
Supplement: Additional file 3: Table S2 — Flavopiridol interacting residues with CDK7 wild type and mutant type structures. [file 1479-7364-7-10-S3.doc]

**Supplementary Table S2.** Flavopiridol interacting residues on CDK7 native and mutant models.

| **Native** | **I63R** | **H135R** | **T285M** |
| --- | --- | --- | --- |
| GLY21 | HIS71 | HIS71 | HIS71 |
| GLN22 | PRO72 | PRO72 | PRO72 |
| PHE23 | GLN123 | ASN73 | GLN123 |
| ALA24 | GLU126 | GLN123 | GLU126 |
| VAL26 | TYR127 | GLU126 | TYR127 |
| LYS41 | GLN130 | TYR127 | GLN130 |
| PHE91 |  | GLN130 |  |
| ASP97 |  |  |  |
| ASN141 |  |  |  |
| LEU144 |  |  |  |
| ALA154 |  |  |  |
| SER161 |  |  |  |
